# Analysis of failed therapy evaluations in radioembolization of primary and secondary liver cancers

**DOI:** 10.1007/s00432-020-03443-z

**Published:** 2020-11-06

**Authors:** Max Masthoff, Philipp Schindler, Fabian Harders, Walter Heindel, Christian Wilms, Hartmut H. Schmidt, Andreas Pascher, Lars Stegger, Kambiz Rahbar, Michael Köhler, Moritz Wildgruber

**Affiliations:** 1 Institute of Clinical Radiology, University Hospital Muenster, Albert-Schweitzer-Campus 1, 48149 Munster, Germany; 2 Department of Gastroenterology and Hepatology, University Hospital Muenster, Muenster, Germany; 3 Department for General, Visceral and Transplantation Surgery, University Hospital Muenster, Muenster, Germany; 4 Department of Nuclear Medicine, University Hospital Muenster, Muenster, Germany; 5 Klinik und Poliklinik für Radiologie, Klinikum Der Universität München, Munich, Germany

**Keywords:** ^99m^Tc-macroaggregated albumin, Pretreatment evaluation, Radioembolization, Liver cancer

## Abstract

**Purpose:**

To analyze patients’ characteristics and reasons for not performing planned transarterial radioembolization (TARE) in liver cancer after ^99m^Tc-labeled macroaggregated albumin (^99m^Tc-MAA) evaluation.

**Methods:**

In this retrospective single-center cohort, all patients undergoing ^99m^Tc-MAA evaluation prior to planned TARE for primary or secondary liver cancer between 2009 and 2018 were analyzed. Patients were assigned to either “TARE” or “no TARE” group. Patients’ characteristics, arising reasons for not performing the planned TARE treatment as well as predictive factors for occurrence of these causes were analyzed.

**Results:**

436 patients [male = 248, female = 188, median age 62 (23–88) years] with ^99m^Tc-MAA evaluation prior to planned TARE of primary or secondary liver cancer were included in this study. 148 patients (33.9%) did not receive planned TARE. Patients with a hepatic tumor burden > 50%, no liver cirrhosis, no previous therapies and a higher bilirubin were significantly more frequent in “no TARE” compared to “TARE” group. Main reasons for not performing TARE were extrahepatic tracer accumulation (*n* = 70, 40.5%), non-target accumulation of ^99m^Tc-MAA (*n* = 27, 15.6%) or a hepatopulmonary shunt fraction of more than 20% (*n* = 23, 13.3%). Independent preprocedural parameters for not performing planned TARE were elevated bilirubin (*p* = 0.021) and creatinine (*p* = 0.018) and lower MELD score (*p* = 0.031).

**Conclusion:**

A substantial number of patients are precluded from TARE following ^99m^Tc-MAA evaluation, which is, therefore, implicitly needed to determine contraindications to TARE and should not be refrained from in pretreatment process. However, a preceding careful patient selection is needed especially in patients with high hepatic tumor burden and alteration in lab parameters.

## Background

Transarterial radioembolization (TARE) with ^90^Yttrium (^90^Y)-loaded microspheres is an increasingly applied treatment option in primary and secondary liver cancers (Mahnken 2016). Recent studies have shown a variable value of TARE in liver cancer. On the one hand, no benefit of overall survival of first-line TARE added to chemotherapy for metastatic colorectal cancer (mCRC) (Wasan et al. 2017) or of TARE added to Sorafenib for hepatocellular carcinoma (HCC) treatment (Ricke et al. 2019) was reported, but on the other hand TARE showed beneficial results in advanced HCC, intrahepatic cholangiocarcinoma or colorectal cancer metastasis of distinct origin (Gibbs et al. 2018; Hoffmann et al. 2012; Klompenhouwer et al. 2017; Kohler et al. 2019). While TARE has been reported to be a safe method with low rates of periprocedural complications, adverse events due to toxicity have been reported including gastrointestinal ulcers, cytopenia, post-embolization syndrome (fatigue, fever, pain, nausea), a decrease in liver function or radioembolization-induced liver disease (REILD; ascites, hepatic insufficiency, jaundice) (Klompenhouwer et al. 2017; Benson et al. 2013; Bester et al. 2013). In this context, angiographically administered ^99m^Tc-labeled macroaggregated albumin (^99m^Tc-MAA) is used to mimic accumulation and distribution of ^90^Y-microspheres during TARE. It is thereby an established pre-therapeutic evaluation tool to predict tumor accumulation of ^90^Y-microspheres as well as to reveal severe lung or gastrointestinal shunting prior to therapy to reduce the risk of adverse advents during TARE. Most studies regarding the value of ^99m^Tc-MAA evaluation and TARE therapy only report about patients with realized TARE. Data on patients who have not been treated with TARE after initial ^99m^Tc-MAA evaluation and related reasons especially in advanced liver cancer are limited.

We, therefore, aimed to (1) evaluate the incidence and the underlying reasons for not performing TARE after ^99m^Tc-MAA evaluation in advanced liver cancer and (2) identify independent preprocedural baseline parameters predictive for not performing a TARE after ^99m^Tc-MAA evaluation or for any of the determined underlying reasons.

## Methods

### Study design

The study was carried out as a retrospective single-center observational trial in a tertiary care academic medical center. The study was approved by the local ethics committee of the Westfälische Wilhelms-Universität Münster, Germany (protocol number 2018-638-f-S). Informed consent was waived due to the retrospective character of the study. This study was performed in accordance with the ethical standards in the 1964 Declaration of Helsinki and its later amendments.

### Patient selection

All subsequent patients with primary or secondary liver cancer undergoing ^99m^Tc-MAA evaluation prior to planned TARE in our center between 2009 and 2018 were included in this study. All patients undergoing MAA scan had approval of the interdisciplinary gastrointestinal tumor board after diagnosis was made based on the according European guidelines (Galle et al. 2018; Oberg et al. 2012; Senkus et al. 2015; Valle et al. 2016; Cutsem et al. 2016). Assignment to ^99m^Tc-MAA evaluation was dependent on a sufficient general condition, an adequate hepatic function (Child–Pugh liver function grade A or B; alanine aminotransferase (ALT) and aspartate aminotransferase (AST) ≤ 5 × upper limit of normal (ULN); total bilirubin ≤ 1.5 ULN; albumin ≥ 29 g/l) as well as adequate hematologic, clotting and renal function tests. Further, life expectancy was supposed to be more than 12 weeks. Demographic patient data, tumor type, previous therapies, hepatic tumor burden and pre-therapeutic bilirubin and creatinine were analyzed.

Assignment to TARE after ^99m^Tc-MAA evaluation was, besides the above-mentioned requirements in patient’s general condition and lab parameters, dependent feasibly to adequately position the catheter, no life-threatening intolerability of the contrast agent, a hepatopulmonary shunt fraction less than 20% and no relevant extrahepatic tracer accumulation. If one of these points was not applicable after ^99m^Tc-MAA evaluation, the planned TARE procedure was terminated.

### Procedure details

Evaluation with ^99m^Tc-MAA was routinely performed in all patients with primary or secondary liver cancer and planned TARE. The angiographic procedures were performed by experienced interventional radiologists who were approved within the quality assurance program of the microsphere provider. Evaluation included embolization of aberrant vessels originating from the hepatic circulation. After ^99m^Tc-MAA application via a coaxial microcatheter system patients underwent planar whole body and SPECT/CT scanning of the thoracic and abdominal region (GE Discovery NM630 or Siemens Symbia T2) using low-energy collimators for dose calculation, detection of extrahepatic tracer accumulation and assessment of hepatopulmonary shunting. While parameters like feasibility to position the catheter and (abnormal) vascular anatomy were rated by interventional radiologists, ^99m^Tc-MAA associated parameters such as hepatopulmonary shunt fraction and extrahepatic tracer accumulation were rated by experienced physicists and nuclear medicine physicians.

^90^Y radioembolization using resin microspheres (SIR-Spheres®; Sirtex Medical, Sydney, Australia) was performed according to standard operating procedures. The ^90^Y dose was calculated based on the body surface area (BSA) method [Activity of SIR-Spheres in GBq = (BSA − 0.2) + (volume of tumor/volume of whole liver)].

### Data collection

All patient and procedural data were retrospectively acquired from the electronic patient’s records as well as from the Picture Archiving and Communications System (PACS).

### Statistical analysis

Data are shown as total number and percentage, mean and standard deviation or median and range or 95% confidence interval (CI), as appropriate. Chi-square test was performed for analysis of TARE vs. no TARE group after ^99m^Tc-MAA evaluation, in case of multiple variables, additional Chi-square test with Bonferroni correction for each pair was applied. Multinomial logistic regression was performed to determine independent prognostic factors that resulted in not treating patients with TARE following each “negative” ^99m^Tc-MAA evaluation. A *p* value < 0.05 was considered statistically significant. Statistical analysis was performed using the SPSS Statistics version 26 (SPSS Inc., Chicago, IL, USA).

## Results

### Characteristics of patients w/o TARE after pretreatment evaluation

436 patients [male = 248, female = 188, median age, years: 62 (23–88)] with ^99m^Tc-MAA evaluation prior to planned TARE were included in this study. Detailed patient characteristics are presented in Table 1. Patients suffered from various types of primary and secondary liver cancers (hepatocellular carcinoma *n* = 120, colorectal cancer *n* = 120, breast cancer *n* = 43, intrahepatic cholangiocarcinoma *n* = 50, metastatic neuroendocrine carcinoma *n* = 20, others *n* = 83). 97 patients (22.2%) had preexisting liver cirrhosis. Hepatic tumor burden was < 25% in 165 patients (37.8%), 25–50% in 224 patients (51.4%) and > 50% in 46 patients (10.6%). 82.6% of the patients (*n* = 360) had undergone previous therapy prior to the planned TARE. Specifically, 63.1% (*n* = 275) had undergone previous chemotherapy and 63.7% (*n* = 278) had received other previous treatments such as surgical resection (25.0%, *n* = 109), external beam radiotherapy (EBRT, 14.2%, *n* = 62) or transarterial chemoembolization (TACE, 11.0%, *n* = 48). Before ^99m^Tc-MAA evaluation, mean total bilirubin was 0.7 ± 0.6 mg/dl, mean creatinine was 0.9 ± 0.3 mg/dl and mean MELD score was 7.8 ± 2.6.

**Table 1:** Patient characteristics

Parameter	Number of patients (%)
All patients	436 (100.0)
Sex	
Male	248 (56.9)
Female	188 (43.1)
Primary tumor	
HCC	120 (27.5)
CrC	120 (27.5)
BrC	43 (9.9)
ICC	50 (11.5)
mNET	20 (4.6)
Others^a^	83 (19.0)
Liver cirrhosis	
Yes	97 (22.2)
No	339 (77.8)
Hepatic tumor burden	
< 25%	165 (37.8)
25–50%	224 (51.4)
> 50%	46 (10.6)
Previous therapy	
Yes	360 (82.6)
No	74 (16.7)
Unknown	2 (0.7)
Previous chemotherapy	
Yes	275 (63.1)
No	150 (34.4)
Unknown	11 (2.5)
Other previous therapies	
Immunotherapy	39 (8.9)
EBRT	62 (14.2)
Operative resection	109 (25.0)
TARE	16 (3.7)
TACE	48 (11.0)
Transplantation	4 (0.9)
Response to previous therapies	
Remission	6 (1.4)
Stable disease	47 (10.8)
Progressive disease	305 (70.0)
No previous therapies	74 (17.0)
Unknown	4 (0.9)
Bilirubin, mean ± SD (mg/dl)	0.7 ± 0.6
Creatinine, mean ± SD (mg/dl)	0.9 ± 0.3
MELD score, mean ± SD	7.8 ± 2.6

*HCC* hepatocellular carcinoma, *CrC* colorectal cancer, *BrC* breast cancer, *ICC* intrahepatic cholangiocarcinoma, *mNET* metastatic neuroendocrine tumor, *EBRT* external beam radiotherapy, *TACE* transarterial chemoembolization, *TARE* transarterial radioembolization, *SD* standard deviation

^a^Pancreas carcinoma (*n* = 15), melanoma (*n* = 14), cancer of unknown primary (*n* = 6), prostate carcinoma (*n* = 7), choroidal melanoma (*n* = 6), lung cancer (*n* = 4), cervical cancer (*n* = 4), small cell lung cancer (*n* = 3), laryngeal carcinoma (*n* = 3), stomach cancer (*n* = 2), leiomyosarcoma (*n* = 2), esophagus carcinoma (*n* = 4), angiosarcoma (*n* = 2), renal cell carcinoma (*n* = 3), parotid carcinoma (*n* = 1), thyroid carcinoma (*n* = 2), thymus carcinoma (*n* = 1), urothelial carcinoma (*n* = 2), Klatskin tumors (*n* = 1), yolk sac carcinoma (*n* = 1)

148 patients (33.9%) with a total number of *n* = 173 ^99m^Tc-MAA evaluations did not proceed to TARE. Detailed characteristics of sub-grouped patients with ^99m^Tc-MAA evaluation with (“TARE” group) and without (“no TARE” group) following TARE are shown in Table 2.

**Table 2:** Patient w/o TARE after ^99m^Tc-MAA evaluation

Parameter	TARE after ^99m^Tc-MAA number of patients (%)	No TARE after ^99m^Tc-MAA number of patients (%)	*p* value		Percentage of patients with no TARE (%)
			Chi-square	Post-hoc chi-square*	
All patients	288 (100.0)	148 (100.0)			33.9
Sex					
Male	169 (58.7)	79 (53.4)	0.290	n/a	31.9
Female	119 (41.3)	69 (45.6)		n/a	36.7
Primary tumor					
HCC	92 (31.9)	28 (18.9)	0.110	0.004	23.3
CrC	73 (25.3)	47 (31.8)		ns	39.2
BrC	25 (8.7)	18 (12.2)		ns	41.9
ICC	32 (11.1)	18 (12.2)		ns	36.0
mNET	13 (4.5)	7 (4.7)		ns	35.0
Others	53 (18.4)^+^	30 (20.3)^++^		ns	36.1
Liver cirrhosis					
Yes	75 (26.0)	22 (14.9)	0.008	n/a	22.7
No	213 (74.0)	126 (85.1)		n/a	37.2
Hepatic tumor burden					
< 25%	121 (42.0)	44 (29.7)		0.014	26.7
25–50%	143 (49.7)	81 (54.7)	0.015	ns	36.2
> 50%	24 (8.3)	22 (14.9)		0.033	47.8
Previous therapy					
Yes	249 (86.5)	111 (76.0)		0.001	30.8
No	39 (13.2)	35 (23.6)	0.001	0.001	47.3
Unknown	0 (0.0)	2 (1.4)		ns	100.0
Previous chemotherapy					
Yes	182 (63.2)	93 (62.8)		ns	33.8
No	101 (35.1)	49 (33.1)	0.339	ns	32.7
Unknown	5 (1.7)	6 (4.1)		ns	54.5
Other previous therapies					
Immunotherapy	28 (9.7)	11 (7.4)	0.043	ns	28.2
EBRT	41 (14.2)	21 (14.2)		ns	33.9
Operative resection	71 (24.7)	38 (25.7)		ns	34.9
TARE	13 (4.5)	3 (2.0)		ns	18.8
TACE	40 (13.9)	8 (5.4)		0.009	16.7
Transplantation	4 (1.4)	0 (0)		ns	0.0
Response to previous therapies					
Remission	4 (1.4)	2 (1.4)		ns	33.3
Stable disease	33 (11.5)	14 (9.5)		ns	29.8
Progressive disease	212 (73.6)	93 (62.8)	0.041	ns	30.5
No previous therapies	39 (13.2)	35 (23.6)		0.004	47.3
Unknown	0 (0.0)	4 (2.7)		ns	100.0
Bilirubin, mean ± SD (mg/dl)	0.7 ± 0.4	0.9 ± 0.8		0.013^#^	
Creatinine, mean ± SD (mg/dl)	0.9 ± 0.3	0.9 ± 0.4		0.345^#^	
MELD score, mean ± SD	7.7 ± 2.5	8.0 ± 3.0		0.384^#^	

*HCC* hepatocellular carcinoma, *CrC* colorectal cancer, *BrC* breast cancer, *ICC* intrahepatic cholangiocarcinoma, *mNET* metastatic neuroendocrine tumor, *EBRT* external beam radiotherapy, *TACE* transarterial chemoembolization, *TARE* transarterial radioembolization, *SD* standard deviation, *ns* not significant, *n/a* not applicable

^+^Pancreas carcinoma (*n* = 12), melanoma (*n* = 8), cancer of unknown primary (*n* = 4), prostate carcinoma (*n* = 4), choroidal melanoma (*n* = 3), lung cancer (*n* = 3), cervical cancer (*n* = 2), small cell lung cancer (*n* = 2), laryngeal carcinoma (*n* = 2), stomach cancer (*n* = 2), leiomyosarcoma (*n* = 2), esophagus carcinoma (*n* = 2), angiosarcoma (*n* = 2), renal cell carcinoma (*n* = 1), parotid carcinoma (*n* = 1), thyroid carcinoma (*n* = 1), thymus carcinoma (*n* = 1), urothelial carcinoma (*n* = 1)

^++^Pancreas carcinoma (*n* = 3), melanoma (*n* = 6), cancer of unknown primary (*n* = 2), prostate carcinoma (*n* = 3), choroid melanoma (*n* = 3), lung cancer (*n* = 1), cervical cancer (*n* = 2), small cell lung cancer (*n* = 1), laryngeal carcinoma (*n* = 1), esophagus carcinoma (*n* = 2), renal cell carcinoma (*n* = 2), thyroid carcinoma (*n* = 1), urothelial carcinoma (*n* = 1), Klatskin tumors (*n* = 1), yolk sac carcinoma (*n* = 1)

*Considering Bonferroni correction

^#^Two-sided students’ *t* test

Within the group of patients not proceeding to TARE, 76.0% (*n* = 111) had previous therapy prior the planned TARE. Here, 62.8% (*n* = 93) had undergone previous chemotherapy and 54.7% (*n* = 81) had undergone other therapies such as surgical resection (25.7%, *n* = 38), EBRT (14.2%, *n* = 21) or TACE (5.4%, *n* = 8). Baseline preprocedural mean total bilirubin was 0.9 ± 0.8 mg/dl, which was significantly higher than in the “TARE” group (0.7 ± 0.4 mg/dl, *p* = 0.013). Mean creatinine was 0.9 ± 0.4 mg/dl and mean MELD score was 8.0 ± 3.0, which was not significantly different from the “TARE” group.

Patients with HCC were significantly lower represented in the “no TARE” (18.9%) than in “TARE” group (31.9%, *p* = 0.004). Thus, the fraction of patients not receiving a TARE after ^99m^Tc-MAA evaluation was lowest in HCC (23.3%) compared to other disease entities of liver cancer (35.0–41.9%). All other disease entities did not show any significant differences between the both groups.

The presence or absence of liver cirrhosis was significantly correlated with proceeding or not proceeding to TARE (*p* = 0.008). Here, 37.2% of the patients with no cirrhosis did not proceed to TARE while this was the case in only 22.7% of the patients with cirrhosis.

The degree of hepatic tumor burden was significantly associated with proceeding or not proceeding to TARE (0.015). Here, patients with a tumor burden of > 50% were significantly more frequent in the “no TARE” group (14.9%) than in the “TARE” group (8.3%, *p* = 0.033) while patients with a tumor burden < 25% where significantly more frequent in the “TARE group” (42.0) than in the “no TARE” group (29.7%, *p* = 0.014). Here, 47.8% of patients with a high tumor burden of > 50% did not proceed to TARE, while this was only the case for 26.7% of patients with a tumor burden < 25%.

The history of previous therapies was as well significantly associated with proceeding or not proceeding to TARE (*p* = 0.001). Patients with no previous therapies were significantly more common in the “no TARE group” (23.6%) than in the “TARE group” (13.2%, *p* = 0.001). Here, 47.3% of patients with no previous therapy did not proceed to TARE after ^99m^Tc-MAA evaluation. Within the group of patients with previous therapies, patients with previous TACE were significantly less frequent in the “no TARE” group (5.4%) than in the “TARE” group (13.9%, *p* = 0.009). The respective fraction of patients not receiving TARE after ^99m^Tc-MAA evaluation was lowest for previous TACE patients (16.7%) and highest for previous EBRT patients (33.9%) and surgical resection (34.9%).

### Preprocedural parameters associated with not proceeding to TARE

Independent baseline parameters as determined prior to ^99m^Tc-MAA evaluation associated with not performing TARE subsequently after pretreatment evaluation were elevated bilirubin (*p* = 0.021) and creatinine (*p* = 0.018) as well as a lower MELD score (*p* = 0.031). All other analyzed parameters such as sex, age, tumor type, hepatic tumor burden, previous therapies or lab parameters such as international normalized ratio (INR), ALT, AST and gamma-glutamyltransferase (yGT) had no predictive value for not performing TARE after ^99m^Tc-MAA evaluation.

### Reasons for not performing TARE after ^99m^Tc-MAA evaluation

The main reason of “negative” ^99m^Tc-MAA evaluation resulting in not performing TARE was extrahepatic tracer accumulation (*n* = 70, 40.5%). In addition, non-target accumulation of ^99m^Tc-MAA (*n* = 27, 15.6%) was one reason not to perform TARE, which only occurred between 2009 and 2015 (see “Discussion” section for further information). Other reasons for not performing TARE were a hepatopulmonary shunt fraction of more than 20% (*n* = 23, 13.3%). An abnormal vascular anatomy without a safe catheter position to securely perform TARE occurred in *n* = 15 (8.7%) evaluations. Other reasons were a deterioration in patients’ general condition (*n* = 16, 9.2%) or liver parameters (*n* = 5, 2.9%) in between ^99m^Tc-MAA evaluation and planned TARE. Non-appearance of the patient (*n* = 8, 4.6%) or intolerance of the contrast agent (*n* = 1, 0.6%) were also observed. In *n* = 8 cases (4.6%), the reason for not performing a TARE was not sufficiently documented.

^99m^Tc-MAA re-evaluation was performed in a total of *n* = 12 patients of the entire study cohort, most of them showing extrahepatic tracer accumulation (*n* = 8/12). Here, in *n* = 5/12 patients (41.7%), TARE was enabled after repeated evaluation by optimized catheter positioning or additional vessel embolization. Regarding patients with prior extrahepatic tracer accumulation, TARE was enabled in *n* = 4/8 patients (50%).

### Preprocedural parameters associated with particular reasons for not proceeding to TARE

Next, we analyzed if any independent baseline preprocedural parameters for the observed reasons not to perform TARE following ^99m^Tc-MAA evaluation could be identified. Here, a higher hepatic tumor burden significantly increased the probability for the occurrence of a non-accumulation of ^99m^Tc-MAA within the tumor (*p* = 0.016) as well as for a deterioration of liver parameters after ^99m^Tc-MAA precluding from TARE (*p* = 0.047). Further, a higher bilirubin (*p* = 0.020) and creatinine (*p* = 0.048) level as well as a lower patient age (*p* = 0.030) was significantly associated with the occurrence of a contraindicatory hepatopulmonary shunt fraction of more than 20%. Further, history of previous therapies (*p* = 0.018) as well as a lower ALT and a higher AST value increased the probability of low patient’s general condition prohibiting the planned TARE. A lower MELD score was associated with the occurrence of extrahepatic ^99m^Tc-MAA tracer accumulation (*p* = 0.020) precluding from TARE. Besides, there were no further independent variables that prevented planned TARE following ^99m^Tc-MAA evaluation.

## Discussion

Within this study, we first analyzed how frequently a planned TARE was not performed after initial ^99m^Tc-MAA evaluation. We found a rate of 33.9% of patients not receiving the planned TARE. In contrast, within the SIRFLOX study only 21 of 267 patients (7.9%), in the FOXFIRE study only 15 of 182 (8.2%) and in the FOXFIRE-Global only 12 of 105 (11.4%) did not receive a TARE after being assigned to the respective treatment group (Wasan et al. 2017; Hazel et al. 2016). In these studies, however, TARE was evaluated as first-line therapy of metastatic colorectal cancer while the patient cohort of our study (1) is more heterogeneous with regards to the underlying primary tumor and (2) suffers from a more advanced stage of cancer disease. In advanced liver cancer, as shown for HCC in the SARAH (22.4%), SIRveNIB (23.1%) or SORAMIC (15.3%) study, drop-out rates from a planned TARE have been reported to be higher (Ricke et al. 2019; Chow et al. 2018; Vilgrain et al. 2017). Moreover, the lower aforementioned numbers compared to our data were observed in dedicated settings of prospective studies, while our data represent a real-life context. In this context, two studies with a more comparable patient cohort report that contraindications to the planned TARE occurred in 22.5% of the patients or that in 29% the therapy plan had to be changed after ^99m^Tc-MAA evaluation (Ahmadzadehfar et al. 2010; Wondergem et al. 2013).

We further analyzed differences of patients’ characteristics within the “TARE” versus the “no TARE” group. Here, patients with HCC as primary tumor, presence of liver cirrhosis, a hepatic tumor burden below 25% and history of previous therapies (especially previous TACE) were significantly less frequent in the “no TARE” group, while patient with a hepatic tumor burden above 50%, no history of previous therapies and a higher bilirubin were significantly more frequent in the “no TARE” group compared to the “TARE” group. This illustrates that patients with a high tumor burden of non-HCC entities in non-cirrhotic livers more probably fail to proceed to TARE after ^99m^Tc-MAA evaluation. Growing experience improving treatment strategies and technical improvements for, e.g., regarding available catheters or approaches for dosimetry recently have and will further reduce the exclusion rate after ^99m^Tc-MAA evaluation. However, further studies investigating the frequency of patients’ preclusion from TARE and according selection criteria as well as technical advancements are needed. In this context, other strategies for planning and evaluation before TARE, such as ^166^Ho (holmium)-based microspheres, may provide novel insights (Smits et al. 2019). Further, initial reports have shown that including cone-beam CT (CBCT) to TARE evaluation and treatment work flow may provide additional information about hepatic tumor burden, tumor and tissue perfusion and extrahepatic enhancement compared to digital subtraction angiography (DSA) or ^99m^Tc-MAA based SPECT/CT imaging (Gormez et al. 2020; Louie et al. 2009; Maleux et al. 2020). However, superiority of CBCT regarding patient safety or treatment evaluation and response has not yet been shown in larger patient cohorts. Moreover, one has to consider a considerably higher radiation dose, especially in case of multiphase and/or repetitive CBCT. Thus, CBCT is currently not routinely performed in every institution.

Second, we analyzed if any independent baseline parameters for not performing the planned TARE after ^99m^Tc-MAA evaluation could be identified. In our study, elevated bilirubin and creatinine as well as a lower MELD score were found to increase the risk for not performing the TARE after ^99m^Tc-MAA evaluation, which may partly be explained by the observation that lab parameters have been associated with the degree of the lung shunt fraction (Kallini et al. 2017). Of notice, to be eligible for ^99m^Tc-MAA evaluation and thereby to be included in this study, total bilirubin had to be ≤ 1.5 ULN. However, our results show that elevation in lab parameters indicating hepatic or renal function above normal values but below common inclusion criteria for TARE increases the risk for not performing TARE.

Third, we analyzed reasons for not performing TARE after ^99m^Tc-MAA evaluation, which currently remain vague in the available literature, and looked if any independent baseline parameters could be associated with those. Here, the main reason not to proceed to TARE after ^99m^Tc-MAA evaluation was extrahepatic tracer accumulation (40.5%), which is in line with other studies finding an extrahepatic accumulation in up to 42% of the examinations (Ahmadzadehfar et al. 2010). Importantly, if applicable, repeating ^99m^Tc-evaluation with optimization of catheter positioning or additional vessel embolization has been shown to reduce the number patients precluded from TARE due to extrahepatic tracer accumulation by roughly 50% (Theysohn et al. 2015), which is in line with results from the presented study. The second most frequent reason, which was only observed between 2009 and 2015, was non-target accumulation of ^99m^Tc-MAA (15.6%). In 2009, when the TARE programme started at our center, the team of interventional radiologists and nuclear medicine physicians were convinced, like many other centers treating patients with TARE, that a TARE in case of non-accumulation of ^99m^Tc-MAA within the tumor target would be of no benefit to the patient. A study published in 2015 showed instead that patients with a low tumor uptake on pre-therapeutic ^99m^Tc-MAA imaging should not be excluded from TARE due to a higher sphere uptake in 60% of the cases, changing existing treatment concepts at that time (Ilhan et al. 2015). Another observed contraindication for TARE after ^99m^Tc-MAA evaluation was a hepatopulmonary shunt fraction of more than 20%, which was observed in 13.3% of the patients. Other studies found such a high lung shunt in 4–9.1% of the patients (Kallini et al. 2017; Bailey et al. 2017). In our study, a younger patient age was significantly associated with a hepatopulmonary shunt fraction of more than 20%, which may be explained by a higher capacity to develop shunts in these patients. Other studies found increased albumin above normal and the presence of macrovascular invasion to be associated with elevated shunt fractions in a study of HCC patients (Kallini et al. 2017). However, our study shows that besides these three common contraindications, there is a not to be neglected amount of further reasons for not performing a TARE occurring at the time or after ^99m^Tc-MAA evaluation, which were observed in 30.6% of patients not proceeding to TARE. Reasons like abnormal vascular anatomy will not be possible to be fully discovered by prior adequate non-invasive imaging techniques. In our study, 4.8% of the entire patient cohort considerably worsen due to rapid tumor progress, deterioration of general condition or laboratory parameters during the time between ^99m^Tc-MAA evaluation and planned TARE contraindicating subsequent radioembolization. This was significantly more probable with a history of previous anticancer treatment, preprocedural alteration of liver parameters or a higher hepatic tumor burden. However, improved baseline assessment and selection of patients before ^99m^Tc-MAA evaluation may help to avoid such courses.

This study is subject to limitations such as its retrospective character and the heterogeneity of the patient cohort, which may influence the incidence of particular contraindications to TARE.

## Conclusions

In conclusion, our study shows that a considerable number of patients are precluded from TARE following ^99m^Tc-MAA evaluation. Non-HCC tumors, a high tumor burden as well as patients without liver cirrhosis and no previous therapies were significantly more frequent in case the planned TARE was not performed. Main reasons for not performing TARE are extrahepatic tracer accumulation and hepatopulmonary shunt fraction of more than 20%. Although we did not identify baseline characteristics or parameters that solitary and unambiguously predict either non-realization of a planned TARE after ^99m^Tc-MAA evaluation or any particular contraindicatory reasons, we show that preprocedural hepatic tumor burden or alteration of lab parameters increases the risk for not performing TARE. Hence, ^99m^Tc-MAA evaluation but also careful patient assessment and selection even before ^99m^Tc-MAA evaluation is implicitly needed to determine common contraindications to TARE but also to reduce the high number of performing pretreatment evaluations without proceeding to TARE.

## Data Availability

Data will be shared by the corresponding author upon reasonable request.

## Abbreviations

^166^Ho: Holmium
^90^Y: Yttrium
^99m^Tc: ^99M^Technetium
ALT: Alanine transaminase
AST: Aspartate transaminase
Bq: Becquerel
BrC: Breast cancer
BSA: Body surface area
CI: Confidence interval
CT: Computer tomography
HCC: Hepatocellular carcinoma
ICC: Intrahepatic cholangiocarcinoma
INR: International normalized ratio
MAA: Macroaggregated albumin
mCRC: Metastatic colorectal cancer
MELD: Model for end-stage liver disease
mNET: Metastatic neuroendocrine tumor
REILD: Radioembolization-induced liver disease
SD: Standard deviation
SPECT: Single-photon emission computed tomography
TACE: Transarterial chemoembolization
TARE: Transarterial radioembolization
ULN: Upper limit of normal
w/o: With or without
yGT: Gamma-glutamyltransferase
